# Protocol for the application of single-cell damage in murine intestinal organoid models

**DOI:** 10.1016/j.xpro.2024.103153

**Published:** 2024-07-30

**Authors:** Anna Elisabeth Seidler, Sören Donath, Lara Gentemann, Manuela Buettner, Alexander Heisterkamp, Stefan Kalies

**Affiliations:** 1 Institute of Quantum Optics, Leibniz University Hannover, 30167 Hannover, Germany; 2 Lower Saxony Center for Biomedical Engineering, Implant Research and Development (NIFE), 30625 Hannover, Germany; 3 REBIRTH Research Center for Translational Regenerative Medicine, 30625 Hannover, Germany; 4 Institute for Laboratory Animal Science, Hannover Medical School, 30625 Hannover, Germany

**Keywords:** Biophysics, Single Cell, Microscopy, Molecular Biology, Organoids

## Abstract

Spatially defined organoid damage enables the study of cellular repair processes. However, capturing dynamic events in living tissues is technically challenging. Here, we present a protocol for the application of single-cell damage in intestinal organoid models. We describe steps for isolating and cultivating murine colon organoids, lentivirus generation and transduction of organoids, single-cell ablation by a femtosecond laser, and follow-up imaging analysis. We provide examples for the image acquisition pipeline of dynamic processes in organoids using a confocal microscope.

For complete details on the use and execution of this protocol, please refer to Donath et al.^1^^,^^2^

## Highlights

• Instructions for the isolation, cultivation, and transduction of intestinal organoids
• Guidelines for the design of lentiviral transfer plasmids suitable for your research
• Precise single-cell ablation in 3D organoids with a femtosecond laser
• Confocal imaging of dynamic wound healing processes in intestinal organoids

## Abstract (editor highlights)

Publisher’s note: Undertaking any experimental protocol requires adherence to local institutional guidelines for laboratory safety and ethics.

## Graphical abstract

**Figure:**
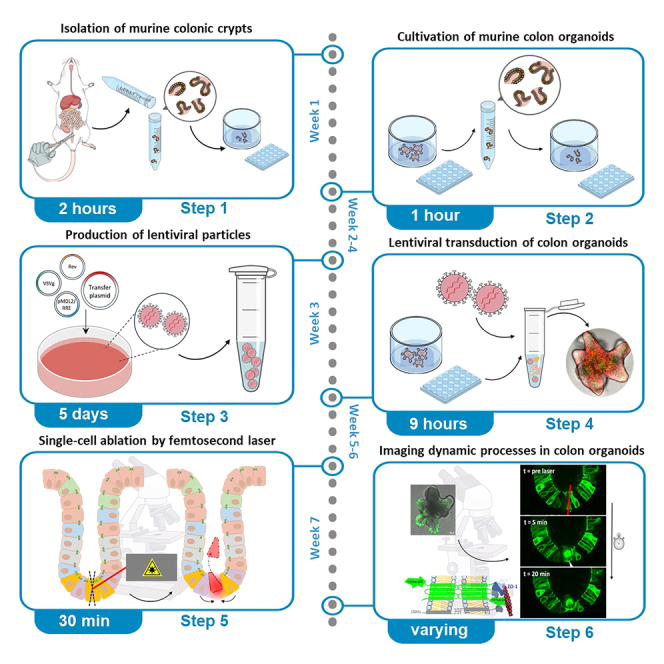

## Before you begin

This protocol demonstrates how to generate highly localized wounding in murine colon organoids via a femtosecond laser system and subsequent live visualization of dynamic processes during intestinal regeneration. However, we also applied the ablation process on murine ileal organoids (Figure S1) and together with the transduction protocol, imaging procedure and analyses even to the study of airway epithelial regeneration using airway organoids.^3^ Visualization is achieved by the genomic introduction of fluorophore-containing peptides via lentiviral transduction. Imaging and readout are described using a confocal microscope and AI-supported analysis of cell parameters by fusion protein Occludin-mEmerald-expressing cells.

***Note:*** This protocol describes a pipeline of experiments, which means that it extends over at least 6–7 weeks in its entirety and thus requires respective time management. The protocol is greatly shortened in the case of already running organoid cultures or prepared virus particles.

### Authorizations

The handling of mice for the murine colonic crypt isolation as well as the work with lentiviruses and class 4 lasers requires you to have following authorization.

1. Your institution requires the approval of the respective responsible authority for performing terminal mice experiments.
2. You need to have access to biosafety level S2 laboratories and permission to perform genetical engineering of mammalian organisms by the local regulatory authority responsible for overseeing work with genetically engineered organisms.
3. You need to be authorized to work with class 4 laser equipment.

### Institutional permissions

4. Animal experiments: This protocol describes the isolation, cultivation, lentiviral transduction, and imaging of murine intestinal organoids. Intestinal crypts were isolated from C57BL/6J wild-type (wt) mice at the age of 6–14 weeks and of both sexes for this purpose. All the experiments were conducted in accordance with the German Animal Welfare Act (§4, TierSchG) after approval by the local institutional advisory committee for animal care and research as well as by the Lower Saxony State Office for Consumer Protection and Food Safety (file number 42500/1H).
5. S2 Laboratory work: The use of specific lentivirus for transduction of murine cells for the visualization of cellular processes in 3D intestinal organoids in compliance with the biosafety level S2 regulations was authorized by the Hannover State Trade Supervisory Office.

### Design of your transfer plasmid for lentiviral transduction

**Timing: 1–2 h**

The transfer plasmid carrying the inserted transgene should be carefully planned and oriented towards the processes to be investigated during wound healing in the intestinal organoid.

**CRITICAL:** The most important things to consider are the total size of the transfer plasmid (should ideally not exceed 10 kb - we have had success with up to 11 kb) and the toxicity of the transgene.

6. Select a suitable 3^rd^ generation lentiviral backbone carrying all required elements for successful lentivirus assembly and genomic insertion in the host genome (e.g., 5′ long terminal repeat (LTR) and 3′LTR, cppT element, WPRE site, HIV-1 Ψ element, HIV-1 Rev responsive element (RRE)).

***Note:*** It can simplify the work considerably if you acquire a lentiviral backbone that already carries the desired prokaryotic and eukaryotic antibiotic gene with the corresponding pro- and eukaryotic promoters upstream.

***Note:*** It makes your work easier if you purchase a backbone that already carries part of your transgene, for example the reporter gene or the desired promoter.

7. Choose a promoter upstream of your transgene between the LTRs suited for your process under investigation.
  a. Use a constitutive promoter if your goal is to express an indicator, like for example the calcium indicator GCaMP, or a (fluorophore-) labeled protein to trace its trafficking or localization during a certain cellular process.
    ***Note:*** It's best to use a promoter that ensures cell type independent and constitutive activity like the CMV, EF1α, UbC, or the PKG promoters. The CMV promoter is very widespread but tends to be silenced in colon organoids. The EF1α promoter is larger, but less susceptible to silencing.^4^
  b. Use an inducible promoter if you require a controlled expression of the transgene, for example to mimic the onset of a disease, various inducible gene expression systems are available.
    ***Note:*** The most commonly used is the tetracycline-induced promoter; it allows a specific and reversible induction or inhibition of transgene expression by placing the TRE promoter upstream of your transgene and controlling its activity by the corresponding tetracycline repressor (TetR) that binds substituted Tet/Dox.^5^
    ***Note:*** These promoter systems can quickly become quite large and go beyond the scope of lentiviral genomes.
  c. Use a specific promoter if your question is, for example, to determine the spatiotemporal resolution of a signaling pathway activity, then signaling pathway-specific promoters are suitable.
    ***Note:*** To obtain signal amplification when your signaling activity might be weak, it is recommended to include several binding sites of the transcription factor in the promoter or, if the activity of the analyzed process is reflected in various transcription factor activities, have multiple different binding sites in the promoter sequence. Here it’s advised to look into literature for already established synthetic protomers. Also, important downstream enhancer sites might need consideration.
    ***Note:*** You should keep in mind that this can interfere with the balance of the intrinsic signaling pathway and alter the physiology of your biological system.
8. Select a reporter gene suited for the visualization of your process of interest utilizing fluorescence and with the most precise spatiotemporal resolution.
  a. You can clone a fluorescent protein downstream of the gene or promoter of interest. This is convenient when you want to study protein localization or signaling activity.
    **CRITICAL:** The fusion of a fluorescent protein to your gene of interest or its lentiviral overexpression can sterically interfere and alter protein folding, interaction, and localization, or disturb the physiological function, for example, when investigating localization, an immunostaining experiment should serve as a control.
    ***Note:*** If you are integrating your gene of interest (GOI) and the fluorescent tag is only supposed to indicate a successful expression of the GOI, it is favorable to separate the GOI and fluorescent protein by an IRES or 2A variant, resulting in individual mRNA strands for each peptide.^6^
    ***Note:*** When using a fluorescent protein downstream of your signaling-sensitive promoter, it is important to think about the strength and period of the respective signaling. Weak signaling activities require fluorescent proteins with high quantum yields, brightness, and high photostability as e.g. mNeonGreen, mStayGold, or mScarlet3.^7^^,^^8^^,^^9^ When your reaction time of signaling activity is of interest, destabilizing domains can shorten the lifetime of the fluorophore, increasing the temporal resolution.
    ***Note:*** Regarding the temporal resolution, however, it is important to note that fluorescent peptide transcription, translation, and maturation are time-consuming processes.
  b. Choose a small peptide tag when struggling with larger fluorescent tags, to allow evasion of steric hindrance.
    ***Note:*** These small tags require the addition of exogenous fluorophores, but not necessarily the permeabilization of the samples. For example, does the tetracysteine tag CCXXCC (18 bp) complex the fluorescein arsenical hairpin binder and generates a strong fluorescence signal.^10^ Also, the SNAP (546 bp; 20 kD)- or CLIP-tag technology from NEB is associated with a high fluorescence yield and, through the commercialization of these systems, also allows the fluorophores to be changed, which is associated with more flexibility when wanting to visualize more than one protein at once.^11^ Small peptide tags such as the SUN-tag can also be a solution when investigating weak signaling activities. Here, 10–24 copies of the short epitope GCN4 can interact with scFV GFP, amplifying the fluorescence response.^12^ These systems can therefore be used if it is known when the protein of interest is expressed, and if you are primarily interested in tracking or interaction studies over a period of hours to a few days.
9. Chose antibiotic selection genes for pro- and eukaryotic selection.

***Note:*** As mentioned in the backbone section, it is advantageous to have two different resistance genes - for prokaryotic and eukaryotic selection - and their respective promoters upstream. Prokaryotic selection can classically be achieved by the β-lactamase (ampicillin/carbenicillin - the latter is more stable and would be recommended if the costs are expandable) downstream of the AmpR promoter. Popular resistance genes for eukaryotic selection are for example puromycin, blasticidin, neomycin/G418 downstream of ubiquitously active promoters as e.g. SV40 or PgK-1.

***Note:*** You can combine the pro- and eukaryotic selection marker when using for example the aminoglycoside phosphotransferase gene from Transposon Tn5 which enables neomycin resistance and having a pro- and eukaryotic promoter upstream in case of total plasmid length problems.^13^

***Note:*** It’s advantageous to choose a backbone plasmid that already contains the resistance genes and associated promoters.

10. Consider inserting small additional elements when designing your transfer plasmid fitting your question, for example, stabilization of the GOI mRNA by a Poly-A site downstream of the GOI sequence, controlling the expression of several genes through the activity of one promoter (bicistronic vector - IRES/2A site), or controlling the localization of the GOI peptide by e.g., an nuclear localization signal (NLS), nuclear export signal (NES) or membrane labeling (CAAX).

**CRITICAL:** The final length of your viral genome is of utmost importance for the success of virus production and integration. There is a fine line between wanting to accommodate multiple components in a plasmid and minimizing promoters or dispensing with enhancer sequences. Based on our experience, total transfer plasmid sizes of up to 11 kb can lead to successful lentiviral transduction of murine intestinal stem cells.

## Key resources table

**Table:** 

REAGENT or RESOURCE	SOURCE	IDENTIFIER
**Bacterial and virus strains**		

NEB 10-beta competent *E. coli* (high efficiency)	NEB	Cat# C3019I
pLV-CMV-Occludin-mEmerald-G418	Donath et al.^2^	N/A
pLV-GCamp5-Blast	Donath et al.^2^	N/A
pLV-7Gli-EGFP-Puro	This paper	N/A
pLV-7Tcf-EGFP-Puro	Fuerer et al.^14^	Addgene plasmid #24305

**Biological samples**		

Murine colonic tissue	Medical School Hannover, ZTL	N/A

**Chemicals, peptides, and recombinant proteins**		

B27 supplement	Invitrogen	Cat# 17504044
BSA	Sigma-Aldrich	Cat# A7030
Chir99021	Hoelzel-Biotech	Cat# USB-C4137
Carbenicillin disodium salt	Carl Roth	Cat# 6344.2
Dextrose	Carl Roth	Cat# 6780.1
DPBS	Sigma-Aldrich	Cat# D8537
EDTA	Carl Roth	Cat# CN06.3
HEPES	Carl Roth	Cat# 6763.1
KCl	Carl Roth	Cat# P017.1
LB-medium (Luria/Miller)	Carl Roth	Cat# X968.2
mEGF	Sigma-Aldrich	Cat# E5160
N-2 supplement	Invitrogen	Cat# 17502048
Na_2_HPO_4_·2H_2_O	Carl Roth	Cat# 4984.1
NaCl	Carl Roth	Cat# HN00.2
Polybrene	Bio-Techne	Cat# 7711/10
Sorbitol	Carl Roth	Cat# 6213.1
Sucrose	Carl Roth	Cat# 4661.1
Tris-HCl	Carl Roth	Cat# 9090.3
TrypLE Select (1×)	Thermo Fisher Scientific	Cat# 12563011
Y-27632	Hoelzel-Biotech	Cat# HY-10583
ZellShield	Minerva Biolabs	Cat# 13-0050

**Experimental models: Cell lines**		

HEK293T	DSMZ	Cat# ACC 635
L-WRN	ATCC	CRL-3276

**Recombinant DNA**		

Plasmid: Occludin-mEmerald-G418	Donath et al.^2^	N/A
Plasmid: GCamp5-Blast	Donath et al.^2^	N/A
Plasmid: H2A-mCherry-Neo	Donath et al.^1^	N/A
Plasmid: 7Gli-EGFP-Puro	This paper	N/A
Plasmid: 7TGP	Fuerer et al.^14^	Addgene plasmid # 24305
Plasmid: pMD2.G	pMD2.G was a gift from Didier Trono (Addgene plasmid # 12259; http://n2t.net/addgene:12259; RRID:Addgene_12259)	Addgene plasmid # 12259
Plasmid: pMDLg/pRRE	Dull et al.^15^	Addgene plasmid # 12251
Plasmid: pRSV-Rev	Dull et al.^15^	Addgene plasmid # 12253

**Software and algorithms**		

Cellpose 2.0	Stringer et al.^16^	https://github.com/MouseLand/cellpose
CellSurgeon	LaserLabSolutions Rowiak	N/A
ImageJ	Schindelin et al.^17^	https://imagej.nih.gov/ij/
Leica Application Suite AF software	Leica Microsystems	N/A

**Other**		

10-min Lenti-X GoStix Plus Tests	Takara	Cat # 631280
15 mL canonical tube	Sarstedt	Cat# 62.554.502
50 mL canonical tube	Sarstedt	Cat# 62.547.254
Achromatic half-wave plate, 690–1,200 nm	Thorlabs	Cat# AHWP05M-950
Axio Observer.D1 inverted microscope	Carl Zeiss AG	N/A
Cell culture flasks, T-175	Sarstedt	Cat# 83.3912.002
Cell culture plates, 24-well	Sarstedt	Cat# 83.3922
Centrifuge	N/A	N/A
Chameleon Ultra II - Ti:Sapphire laser	Coherent	N/A
Cultrex reduced growth factor basement membrane extract (BME), type R1	Bio-Techne	Cat 3433-010-R1
Digital piezo amplifier d-Drive 30DV50	Piezosystem Jena	N/A
DMEM, high glucose	Sigma-Aldrich	Cat# D6429
DMEM, high glucose, GlutaMAX supplement, pyruvate	Thermo Fisher Scientific	Cat# 31966021
DpnI restriction enzyme	Jena Bioscience	Cat# EN-160S
FCS	Sigma-Aldrich	Cat# F7524
Galvanometer optical scanner 6210H	Cambridge Technology	N/A
Glass bottom dishes 35 mm	ibidi	Cat# 81218-200
GoTaq Long PCR master mix	Promega	Cat# M4021
HC FLUOTAR L 25×/0.95 W VISIR	Leica Microsystems	Cat# 11506375
Inject disposable syringes 5 mL (eccentric)	B. Braun Petzold	Cat# 421110
LD LCI Plan-Apochromat 40×/1.2 objective Imm Korr DIC M27	Carl Zeiss AG	Cat# 420862-9970-000
Leica TCS SP5 confocal microscope	Leica Microsystems	N/A
Monarch PCR & DNA Cleanup Kit	NEB	Cat# T1030S
NEB HiFi DNA Assembly master mix	NEB	Cat# E2621L
Optical beam shutter controller	Thorlabs	Cat# SC10
Petri dishes, 100 mm	Sarstedt	Cat# 83.3902
Photomultiplier tube - R6357	Hamamatsu	N/A
Polarizing beamsplitter cube	Thorlabs	Cat# CCM1-PBS252/M
Q5 High-Fidelity 2× master mix	NEB	Cat# M0492S
Six-position motorized filter wheel for Ø1" (Ø25 mm) optics	Thorlabs	Cat# FW102C
Sterican cannula G 24, 0,55 × 25 mm, violet	B. Braun Petzold	Cat# 311217
Ultracentrifuge	N/A	N/A
ZymoPURE II Plasmid Midiprep Kit	Zymo Research	Cat# D4200
Zyppy Plasmid Miniprep Kit	Zymo Research	Cat# D4019

## Materials and equipment

### Two-photon excitation microscopy for precise single-cell ablation

The two-photon excitation microscope (TPEM) can be used for precise ablation of (sub)cellular structures in 3D models. The ablation is caused by a highly localized low-density electron plasma generated via multiphoton ionization of a tightly focused laser beam from a Chameleon Ultra II Ti:Sapphire-laser capable of 120 fs pulses at a repetition rate of 80 MHz.

**Figure 1:**
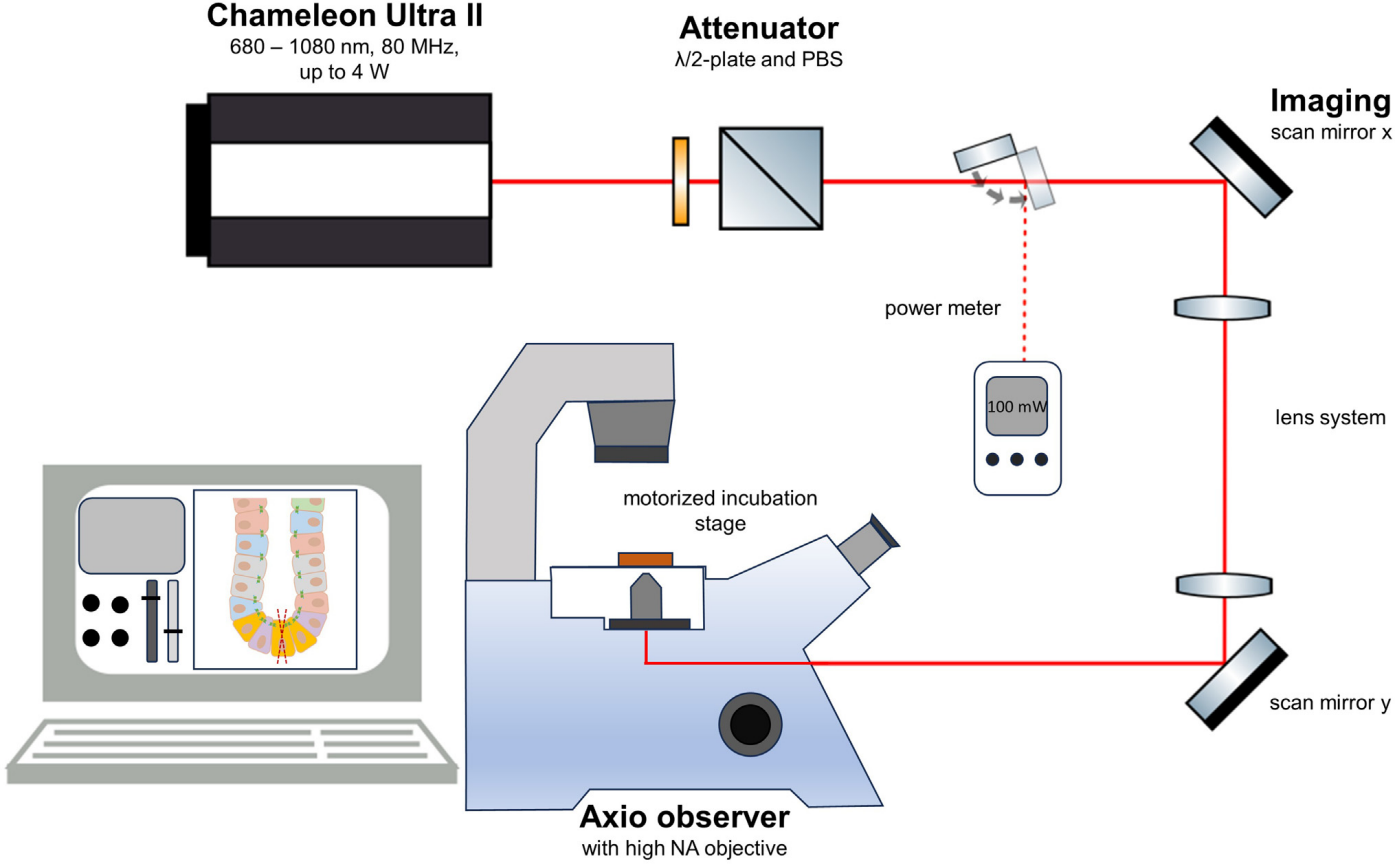
**Schematic representation of the TPEM setup for cellular ablation in 3D colon organoids** The power- and wavelength-adjustable Chameleon Ultra II femtosecond laser generates a laser beam, that is steered into the Zeiss Axio Observer D1 microscope via multiple λ/2-plates, polarizing beam splitters (PBS), and mirror and lens systems. A power meter is used to measure the beam power. The movement of the beam focus point inside the biological sample is achieved by scan mirrors in the x-y-planes and by a piezoelectric system in the z-plane. Laser-induced ablation is performed at a pulse energy of 1.25–3.2 nJ (crypt base) or 1.9–4.4 nJ (matured cell zone).

Setup (illustrated in Figure 1):

The Ti:Sapphire-laser (Chameleon Ultra II) is tunable in the wavelength range of 680–1080 nm. We typically choose 730 nm for cell ablation. The laser power is adjusted by several λ/2-plates and polarizing beam splitters. A shutter controls the exposure time of the laser beam on the biological sample. Visualization is achieved by an integrated Zeiss Axio Observer D1 microscope using a 40× water-immersion objective with a numerical aperture of 1.2. An integrated filter wheel serves for the imaging of different fluorophores and a photomultiplier tube (PMT) detects the optical fluorescence signal. The focal plane is tuned by objective height adjustment via a piezoelectric system (z-plane) and precise cell ablation in the focus plane is enabled by two reflecting galvanometric mirrors regulating the laser beam in x- and y-direction. The system is operated by the custom developed CellSurgeon software. An incubation chamber with 37°C and 5% CO_2_ is installed for long-term experiments to prevent temperature and pH changes in the organoid surrounding.

### Culture media recipes and buffers

• Prepare 1 M HEPES solution, pH 7.5. Sterilize by filtration through 0.45 μm nitrocellulose filter. Store at 4°C for up to 12 months under sterile and well-sealed conditions.
• Prepare 2.5 M CaCl_2_ solution. Sterilize by filtration through 0.45 μm nitrocellulose filter. Store at 4°C for up to 12 months under sterile and well-sealed conditions.
• Prepare 50 mM sodium butyrate solution. Sterilize by filtration through 0.45 μm nitrocellulose filter. Store at 4°C for up to 2 days under sterile and well-sealed conditions.

**Table:** Dissection Buffer

Reagent	Final concentration	Amount
Sucrose	43.3 mM	3 g
D-Sorbitol	54.9 mM	2 g
**Total in 1× PBS**	**N/A**	**200 mL**

Sterilize by filtration through 0.45 μm nitrocellulose filter.

Store at 4°C for up to 6 months under sterile and well-sealed conditions.

**Table:** Organoid Media

Reagent	Final concentration	Amount
DMEM: high glucose, GlutaMAX, pyruvate	50%	22.945 mL
L-WRN-supernatant (L-WRN in DMEM, high glucose, GlutaMAX, pyruvate plus 10% fetal calf serum)	50%	25 mL
N2 (100×)	1×	500 μL
B27 (50×)	1×	1 mL
Recombinant mouse epidermal growth factor (0.5 mg/mL)	50 ng/mL	5 μL
Y-27632 (10 mM)	10 μM	50 μL
Cellshield (100×)	1×	500 μL
**Total**	**N/A**	**50 mL**

Store at 4°C for up to 2 weeks under sterile and well-sealed conditions.

**Note:** There is also commercially available media for the culture of murine organoids, as for example from Stem Cell Technologies. However, the use of these media is associated with increased costs.

**Table:** Cell Culture Media

Reagent	Final concentration	Amount
DMEM: high glucose	85%	445 mL
FBS	10%	50 mL
Penicillin/ Streptomycin	100 U/mL penicillin/100 μg/mL streptomycin	5 mL
**Total**	**N/A**	**500 mL**

Store at 4°C for up to 1 month under sterile and well-sealed conditions.

**Table:** 2× HeBS

Reagent	Final concentration	Amount
Dextrose	12 mM	216.19 mg
HEPES (1 M)	50 mM	5 mL
KCl	10 mM	74.55 mg
NaCl	280 mM	1.636 g
Na_2_HPO_4_·2H_2_O	1.5 mM	26.7 mg
**Total**	**N/A**	**100 mL**

Adjust pH to 7.05.

Sterilize by filtration through 0.45 μm nitrocellulose filter.

Store at 4°C for up to 12 months under sterile and well-sealed conditions.

**Table:** Sucrose Cushion Solution

Reagent	Final concentration	Amount
Tris-HCl (pH 7.4)	50 mM	788 mg
NaCl	100 mM	584.4 mg
EDTA (0.5 M), pH 8.0	0.5 mM	100 μL
Sucrose	20%	20 g
**Total**	**N/A**	**100 mL**

Sterilize by filtration through 0.45 μm nitrocellulose filter.

Store at 4°C for up to 12 months under sterile and well-sealed conditions.

**Table:** Transduction Organoid Media

Reagent	Final concentration	Amount
Organoid Media		1.994 – x mL
Lentivirus in organoid media	approx. 2–4 × 10^7^ IU	x
Polybrene (8 mg/mL)	8 μg/mL	2 μL
CHIR99021 (2.5 mM)	2.5 μM	2 μL
mEGF (100 μg/mL)	100 ng/mL	2 μL
**Total**	**N/A**	**2 mL**

Store at 4°C for up to 2 weeks under sterile and well-sealed conditions.

## Step-by-step method details

### Isolation of murine colonic crypts for organoid culture

**Timing: approximately 2 h**

The discovery of the self-organization of LGR5^+^ intestinal stem cells into 3D organoids when embedded in a supporting extracellular matrix and supplied with appropriate growth factors was made by Clevers and colleagues in 2009 and revolutionized classical *in vitro* cell culture.^18^ The cellular heterogeneity and epithelial interaction make intestinal organoids uniquely suited for studying the epithelial restitution process.^1^^,^^19^ The necessary stem cells are residing in the crypt bases of the intestinal tissue and can be obtained from adult animals by extracting the colon and crypt preparation by EDTA chelation as described in the following step-by-step protocol based on the protocol from Mahe and colleagues in 2013.^20^

1. Precool the centrifuge to 4°C and pre-incubate a 24-well plate at 37°C.
2. Sacrifice a C57BL/6J mouse (age of 6–14 weeks) by CO_2_ overdose followed by cervical dislocation.
  a. Ensure disconnection of the spinal cord and scull by feeling with your fingers.
3. Remove the distal colon.
  a. Place the mouse on the back so the ventral site is accessible.
  b. Disinfect the abdomen with 70% ethanol and open the peritoneum with clean surgical tools.
  c. Locate the colon and remove it from approx. 2–3 cm after the caecum to approx. 1 cm in front of the anus. This results in purely distal colon organoids later on.
    ***Note:*** Try to take as little fatty tissue as possible by slowly pulling the colon out of the peritoneum with blunt forceps and separating attached fat strands.
  d. Remove residual feces by flushing the colon with 10 mL cold 1× PBS using a 10 mL syringe and placing the colon to its opening.
  e. Place the flushed colon in 1× PBS on ice.
  f. Remove residual fat attached to the colon.
4. Prepare the colon into digestible pieces.
  a. Cut the colon lengthwise and clean from the apical side with cold 1× PBS if needed.
  b. Cut the colon in 0.5 cm^2^ pieces and transfer them into 15 mL cold 2 mM EDTA in 1× PBS without Ca^2+^/Mg^2+^ in a 15 mL canonical tube (tube A).
5. Isolate colonic crypts.
  a. Seal the 15 mL tube containing 2 mM EDTA in 1× PBS without Ca^2+^/Mg^2+^ and the colonic tissue pieces with parafilm tightly and place it in a container with ice that’s shaken horizontally for 30 min.
  b. Let the colonic pieces settle to the ground and discard the supernatant.
  c. Quickly wash the colonic tissue pieces with 5 mL 2 mM EDTA in 1× PBS without Ca^2+^/Mg^2+^ twice, slightly swiveling the tube followed by letting the pieces settle to the bottom.
  d. Exchange the supernatant for 5 mL of Dissection buffer (see materials and equipment section) and the 15 mL canonical tube is tightly closed before wrapping it in a paper sheet to protect it from the body heat, and shaken vigorously for 7 min.
    **CRITICAL:** In this step, the crypts are detached from the submucosa; therefore, it should not be shortened and vigorous shaking is of great importance.
  e. Transfer the cloudy supernatant through a 70 μm cell strainer into a new 15 mL canonical tube (tube B).
  f. Shortly wash tube A twice with Dissection buffer and add it through the 70 μm cell strainer to the crypt-containing new tube B.
  g. Centrifuge tube B for 10 min at 500 × *g* at 4°C.
  h. Discard the supernatant and resuspend the pellet in 5 mL cold 1× PBS completely.
  i. Centrifuge for 5 min at 750 × *g* at 4°C. Place the tube on ice.
6. Embed the colonic crypts in the extracellular matrix (here: Cultrex Reduced Growth Factor Basement Membrane Extract (BME), Type R1).
  a. Discard the supernatant and take up the pellet in 0.5–1 mL of 40% organoid media (see materials and equipment section) and 60% Cultrex; resuspend completely but avoid the introduction of air bubbles. Place on ice.
    ***Note:*** The amount of Cultrex:media depends on the length of the distal colon section, as well as the success of the crypt separation. We recommend starting with approx. 500 μL total volume and checking on a slide whether the concentration corresponds to approx. 10–20 crypts/10 μL Cultrex:media solution.
  b. Plate 30 μL crypt-containing Cultrex in three small droplets in each well of a prewarmed 24-well plate.
  c. Let the Cultrex drops solidify for 30 min by placing the plate in an incubator with 37°C and 5% CO_2_.
  d. Add 500 μL prewarmed conditioned organoid media supplemented with additional 10 μM Y-27632 to each well and culture the colon organoid-forming crypts at 37°C with 5% CO_2_.
7. Change media every two days. The increase in Y-27632 concentration from 10 to 20 μM is only maintained in the first two days after isolation.

### Passaging of murine colonic organoids

**Timing: 1 h**

To keep healthy organoids with intact epithelium for the regenerative process, it is important to passage them at correct times. Since most organoid cultures are quite heterogeneous in composition, it is difficult to determine an exact time for passage. Therefore, phenotypic characteristics are described below to determine the correct time.

***Note:*** Generally, size is often a good indicator; organoids between 300 – 600 μm are “fully-grown” and should be used for analysis or passaged.

**Figure 2:**
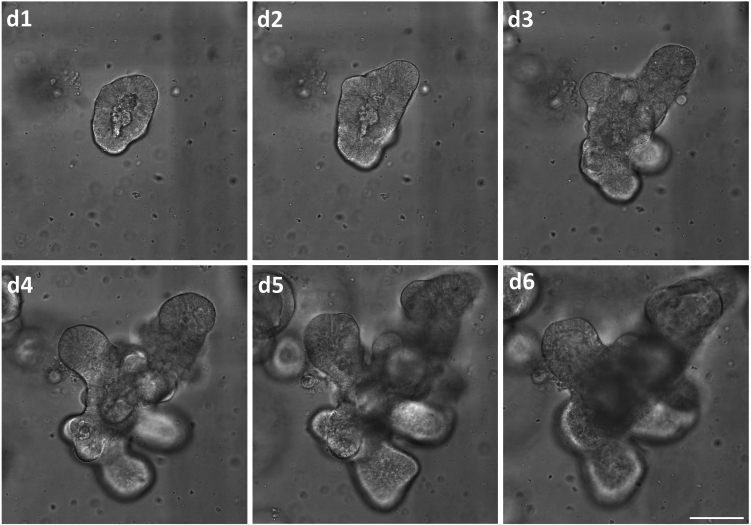
**Bright-field microscope images depicting the growth progression of a representative intestinal organoid over a six-day period after passaging (d1-d6)** The organoid appears closed and roundish at day one (d1) post passaging through a 24G needle four times. Distinct crypts are recognizable from d3 on and the organoid increased in general size and crypt number over the next 2 days. On d6, a darkened lumen and additionally a disruption of the epithelium is already recognizable and passaging was performed immediately. Scale bar = 100 μm.

***Note:*** Intestinal organoids shed dead cells and debris into the apical lumen of the organoids, causing it to darken. These accumulations of cells exert pressure and can, among other things, lead to the rupture of an organoid. Organoids with a darkened lumen should therefore be urgently passaged. In addition to the organoids themselves, brittle Cultrex also indicates the need to passage the culture. These morphologies appear mostly in a range of 5–7 days (Figure 2).

8. Precool the centrifuge to 4°C, pre-incubate a 24-well plate at 37°C, and prewarm organoid media to 37°C.
9. Remove the intestinal organoids from the extracellular matrix.
  a. Place the culture plate on ice to destabilize the extracellular matrix.
  b. Add 500 μL ice-cold 1× PBS to each well to detach the Cultrex-organoid droplets.
  c. Scratch the well bottom with the pipette tip (P1000) and pipette the total volume of 1 mL up and down approx. 5 times to wash residual organoids from the well bottom and dissolve the matrix further.
  d. Collect the organoid-containing solution in a 15 mL canonical tube placed on ice.
  e. Wash the wells with 1 mL 1× PBS and transfer it into the 15 mL canonical tube.
10. Rupture the organoids for propagation purposes.
  a. Pass the organoid solution through a 24G needle three to four times (See Problem 1) to disrupt the organoids into single crypts and dissolve them from the extracellular matrix.
    ***Note:*** The number of passes of the organoids through the 24 G needle depends on the size of the organoids before passaging. Advanced organoids with diameters of approx. 600 μm and 10 or more crypts need passing through the needle approx. 3–5 times while smaller organoids are harder to segment and you need to pass them up to 8 times through the 24 G needle. The organoid example in Figure 2 on d6 should be passed approx. 5 times.
  b. Centrifuge at 750 × *g* at 4°C for 5 min.
  c. Discard the supernatant; first by tilting the canonical tube and then removing the residual liquid using a 100 μL pipette.
11. Embed and plate the organoid fragments in the extracellular matrix.
  a. Take up the pellet in 30 μL Cultrex/well; the number of wells depends on the density of seeded organoids - e.g., if approx. 30 fully developed organoids grew each well, a 1:2 splitting ratio is recommended. And mix thoroughly by carefully pipetting up and down.
  b. Seed 30 μL/well in three droplets and let the Cultrex solidify for 20 min in an incubator at 37°C and 5% CO_2_.
    ***Note:*** The organoid fragments should be distributed evenly and in all levels of the droplets (See Problem 2).
  c. After 20 min add prewarmed 500 μL conditioned organoid media to each well.
12. Change media every two days.

### Generation of the transfer plasmid and production of lentiviruses for organoid transduction

**Timing: approx. 2 weeks**

The use of lentivirus for the fast and stable transduction of eukaryotic cells has the advantage that the permanent integration of viral cDNA into the host genome can also take place in non-dividing cells.

***Note:*** The most commonly used lentiviral packaging system for transgene delivery is based on the HIV-1 virus. To increase the safety of lentivirus use in the lab, your lentiviral transfer plasmid is separated from the packaging and envelop plasmids.

***Note:*** The following protocol refers to the 3^rd^ generation lentiviral packaging system with VSV-G glycoprotein initiating the entry mechanism and HEK293T cells as the lentiviral particle-producing cell line. The packaging plasmid is divided into one encoding for the proteins Gag and Pol (pMDLg/pRRE) and another one encoding for Rev (pRSV-Rev).^15^ The envelope plasmid usually carries the DNA for the VSV-G (pCMV-VSV-G), which enables a broad tropism of the virus. These plasmids are generalized and can be purchased, so you only need to clone the transfer plasmid with the corresponding desired gene of interest inserted into the host genome.

### Generation of the lentiviral transfer plasmid (3^rd^ generation lentiviral packaging system)

**Timing: 2–3 days**

**CRITICAL:** Make sure your primers pairs for the following PCRs (step 14., 19., and 22.) are compatible in terms of annealing temperatures and GC-content. The GC content should not exceed 50–60%.

13. Prepare your lentiviral backbone.
  a. Buy a suitable lentiviral transfer plasmid backbone. They are empty or often provided with a constitutive promoter and common fluorescent protein serving as the transgene sequence.
  b. Digest 3 μg of your lentiviral backbone with restriction enzymes, generating the respective sites for transgene insertion according to the manufactures protocol.

***Note:*** If your lentiviral backbone carries the toxic ccdB gene or the chloramphenicol acetyltransferase gene, it is important to cut these out during digestion in order to ensure growth of the transformed bacteria later or to increase the plasmid yield via chloramphenicol treatment.

**CRITICAL:** Make sure the used restriction enzymes have compatible reaction conditions (See Problem 3).

**CRITICAL:** Make sure your restriction enzymes create sticky ends.

**Pause point:** You can store your cut backbone at −20°C long term.

14. Amplify your transgene by PCR.
  ***Note:*** If your transgene is composed of multiple fragments, then all fragments are first amplified individually by PCR as described in step 14.
  a. Design respective primers to gain the complementary sites to your digested backbone or up- and downstream located additional inserts at the 3′ and 5′ ends of your insert.
  b. Perform PCR using the reaction master mix (Table 1) and cycling conditions (Table 2) provided.

**Table 1:** PCR reaction master mix

Reagent	Amount	Final concentration
DNA template	x	100–500 ng
Q5 High-Fidelity 2× Master Mix	12.5 μL	1×
Forward Primer (10 μM)	1.25 μL	500 nM
Reverse Primer (10 μM)	1.25 μL	500 nM
ddH_2_O	25 μL - x	-

**Table 2:** PCR cycling conditions

Steps	Temperature	Time	Cycles
Initial Denaturation	98°C	30 s	1
Denaturation	98°C	10 s	35 cycles
Annealing	50°C–72°C	30 s	
Extension	72°C	30 s/kb	
Final extension	72°C	2 min	1
Hold	4°C	Forever	

**Note:** Alternative polymerases with their corresponding manufacturer protocols can also be used for amplification of your insert fragments.
15. Digest the template DNA in the PCR product from step 14 using DpnI according to DpnI digestion master mix (Table 3) and cycling conditions (Table 4) provided.

**Table 3:** DpnI digestion master mix

Reagent	Amount	Final concentration
PCR product	25 μL	-
10× Universal Buffer	5 μL	1×
DpnI Restriction enzyme (10 U/μL)	2 μL	20 U
ddH_2_O	23 μL	-

**Table 4:** DpnI digestion cycling conditions

Steps	Temperature	Time
Incubation	37°C	60 min
Heat inactivation	80°C	20 min
16. Check for the correct amplicon length by electrophoresis in 1–2% agarose gel, running at 90 mV for 30–45 min, depending on your amplicon length (See Problem 4).
17. Measure the concentration of your digested lentiviral backbone and amplicon.

***Note:*** Your concentrations are expected to range between 150 – 300 μg/μL for the amplicon and between 0.020–0.100 μg/μL for the cut backbone.

**Pause point:** You can store your amplicon at −20°C.

18. Assemble the final transfer plasmid using the NEB HiFi DNA Assembly Master Mix according to the manufactures protocol.

***Note:*** The amount of insert fragment and backbone DNA used depends on the number of fragments to be assembled.

19. Check the correct assembly of your transfer plasmid by restriction digestion and/or run a PCR over the transgene sequence (See Problem 5).

***Note:*** It’s advised to choose a mix of restriction enzymes and primers cutting or annealing to your backbone as well as the insert.

20. Check for correct length by electrophoresis in a 1–2% agarose gel, running at 90 mV for 30–45 min, depending on your fragments or amplicon length.
21. Introduce the correct transfer plasmid into NEB 10-beta Competent E. coli (High Efficiency) according to the manufacturer’s protocol and cultivated on LB-Agar plates with the respective antibiotic (e.g., 100 μg/mL carbenicillin) for prokaryotic selection of your plasmid-carrying bacteria at 30°C for 16–24 h.
22. The next day, pick 10 colonies and determine effectively transformed clones by colony PCR with primers annealing to the backbone next to the inserted GOI or the second primer in the GOI.
23. Prepare plasmid Minipreps in 5 mL LB medium in 50 mL canonical tubes with the respective antibiotics of the respective clones which showed the desired bands in the gel after colony PCR.
24. Incubate at 37°C for 16–24 h until a clear clouding of the LB medium is recognizable.
25. Perform Minipreps using for example the Zyppy Plasmid Miniprep Kit according to the manufactures protocol.

***Note:*** Process 3 mL of bacterial culture in the first step of the protocol.

26. Prepare 50% glycerol stocks from the successfully grown transformed bacteria solutions and store at −80°C.

***Note:*** It is also possible to wait for the correct sequencing results and store the clone in the refrigerator, but we recommend preparing the glycerol stock as fresh as possible and then sort them out again after sequencing.

27. Sequence at least the transgene segment to exclude mutations and check for correct order of fragments and insertion into the backbone.

**Pause point:** If the glycerol stock is stored at −80 to −150°C, it can be stored for several years.

28. Prepare Midi- or Maxiprep cultures of your clone carrying the correct transfer plasmid for larger quantities of plasmid DNA for lentivirus production according to the manufactures protocol (e.g., ZymoPURE II Plasmid Midiprep Kit).

**Pause point:** Your Plasmid DNA should be stored at −20°C and is stable for several years.

### 3^rd^ generation production of lentiviral particles

**Timing: 5 days**

Lentiviral particles carrying the transgene for visualization of dynamic regenerative processes in intestinal organoids after single-cell ablation can be produced by co-transfecting HEK293T cells with the envelope, packaging, and the individualized transfer plasmid via low-cost calcium-phosphate precipitation, and harvested already two days later. The `sucrose cushion’-step for ultracentrifugation of the lentiviral particles was adapted from Gill and Denham.^21^

### Day −3

#### Prepare HEK293T cells for expansion

**Timing: approx. 15 min**

***Note:*** The HEK293T cell passage number should not exceed P10.

29. Seed 5 × 10^6^ cells/ T175 flask in 30 mL cell culture media (see materials and equipment section). Prepare two T175 flasks.
30. Incubate at 37°C and 5% CO_2_ for three days.

### Day 0

#### Plate HEK293T cells for transfection

**Timing: approx. 30 min**

***Note:*** The HEK293T cells in the T175 flasks should be 90–100% confluent.

31. Collect the HEK293T cells.
  a. Remove the media with a serological pipette and discard it.
  b. Wash the cells with 5 mL/T175 flask prewarmed 1× PBS and dispose the 1× PBS.
  c. Add 5 mL prewarmed Trypsin-0.25% EDTA to each flask and place it in the incubator at 37°C for 3 min so the cells become detached from the flask bottom.
  d. Rinse each flask with 10 mL cell culture media and collect the cell solution in a canonical tube.
  e. Centrifuge for 5 min at 500 × *g*.
32. Count the HEK293T cells.
  a. Take up the cell pellet in 20 mL cell culture media and resuspend thoroughly.
  b. Make a separate 1:10 dilution before counting the cells by using a Neubauer Chamber or Cell counter.
  c. Calculate cell count/mL.
33. Plate HEK293T cells for transfection.
  a. Plate 8 × 100 mm dishes à 6 × 10^6^ cells in 10 mL cell culture media.
  b. Place cells in an incubator with 37°C and 5% CO_2_.

### Day 1

#### Transfecting the HEK293T cells by calcium phosphate precipitation

**Timing: approx. 1–2 h cell culture handling (+2 h pre-incubation time pre transfection and 6 h incubation time post transfection)**

***Note:*** The HEK293T cells should have reached a confluency of 80–90%.

34. Two hours before transfection, change the cell culture media on the HEK293T cells to cell culture media supplemented with 20 mM HEPES and 25 μM chloroquine.

**CRITICAL:** Prevent the detachment of cells from the bottom of the culture dish during the change of medium by using properly prewarmed medium and pipetting carefully and slowly, ideally preparing one dish after another.

***Note:*** Chloroquine neutralizes the pH in lysosomes and thereby inhibits lysosomal DNases and prevents degradation of the plasmid DNA.^22^^,^^23^^,^^24^

35. Transfect HEK293T cells with 3^rd^ generation lentiviral envelope, packaging, and transfer plasmids.
  a. Mix equal numbers of plasmid copies, 1.3 × 10^12^, resulting in 12.5 μg of pMDLg/pRRE (gag/pol), 5.9 μg pRSV (REV), 8.2 μg pMD2.G (VSVg), and x μg of transgene-carrying plasmid per dish, in sterile Millipore water to a total volume of 450 μL/100 mm dish.
  b. Add 50 μL 2.5 M CaCl_2_/100 mm dish to the plasmid mix and vortex the solution.
  c. Slowly drop the DNA:CaCl_2_-solution to 500 μL 2× HeBS buffer (see materials and equipment section)/100 mm dish during constant generation of air bubbles using a sterile 2 mL serological pipette.
  d. Generate air bubbles in the solution for an additional 30 s.
  e. Let the DNA:Ca_3_(PO_4_)_2_ solution sit at 20°C–25°C for 20 min.
  f. Resuspend the co-precipitated plasmid-DNA and add 1 mL/100 mm dish dropwise to the cells.
  g. Incubate the cells for approx. 6 h at 37°C and 5% CO_2_.
36. Stop the transfection process.
  a. After 6 h of incubation, replace the plasmid containing media with 10 mL/100 mm dish cell culture media supplemented with 20 mM HEPES and 1 mM sodium butyrate.

### Day 2

#### Check for successful transfection

(if transgene is suited for visualized confirmation).

37. Check for fluorescence signal if your transgene allows visual confirmation of a successful transfection.

***Note:*** Most fluorophores are already visible 24 h post transfection by a fluorescence microscope.

**CRITICAL:** If you have a signaling pathway-sensitive transgene, make sure your signaling pathway is active in the HEK293T cell line. If you are overexpressing a protein, make sure it is an endogenously expressed protein in HEK293T cells.

### Day 3

#### First virus harvest (48 h post transfection start)

**Timing: 30–40 min**

38. Harvest the virus particle-containing media from the eight 100 mm dishes and collect it in 50 mL canonical tubes.
39. Replace the media with prewarmed 10 mL/100 mm dish cell culture media supplemented with 20 mM HEPES and 1 mM sodium butyrate.
40. Place the HEK293T cells back at 37°C and 5% CO_2_ and incubate for additional 24 h.
41. Centrifuge the collected media at 2000 × *g* for 10 min, preferably at 4°C.
42. Let the supernatant pass through a 0.45 μm sterile filter and store at 4°C for 16–26 h.
43. Preparation for d4: place the rotor for ultracentrifugation at 4°C.

### Day 4

#### Second virus harvest and concentrating (72 h post transfection start)

**Timing: 1–2 h cell culture handling (+ 2 h centrifugation time)**

44. Harvest the virus particle containing media (72 h after transfection).
  a. Harvest the virus particle-containing media from the eight 100 mm dishes and collect it in 50 mL canonical tubes.
  b. Centrifuge the collected media at 2000 × *g* for 10 min, preferably at 4°C.
  c. Let the supernatant pass through a 0.45 μm sterile filter.

***Note:*** The amount of collected virus particle containing media supernatant should be approx. 160 mL in total.

45. Concentrate the virus particles by ultracentrifugation (UC).
  ***Note:*** Keep in mind that every step has to be performed in an S2 lab and under a clean bench – for the next steps you need to place a scale under the clean bench.
  a. Transfer about 32 mL of virus containing media supernatant into a UC tube.
  b. Fill a 5 mL serological pipette up to 7 mL with the sucrose cushion solution (see materials and equipment section) and carefully place the tip of the serological pipette at the bottom of the UC tube and slowly pipette 2 mL “under” the media. Repeat for each UC tube.
  c. Make sure that all the UC tubes have the same weight and adjust with 1× PBS if needed.
  d. Carefully set the UC tubes in their precooled rotor inserts.
  e. Centrifuge at 100.000 × *g* for 2 h at 4°C.
46. Take up the viral particles in organoid media.
  a. Remove the UC tubes from the rotor inserts and discard the supernatant by tilting the tubes.
    ***Note:*** The virus pellet should not be visible to the naked eye, but it often happens that residues not removed by sterile filtration make a milky pellet visible, which, depending on the fluorescent protein, can even take on corresponding colors.
  b. Place the UC tubes upside down on a piece of tissue and leave to dry for 5 min.
  c. Carefully wipe the inside edges of the UC tube with a lint-free tissue to prevent residual media to flow back when placing the tube back on ice.
  d. Absorb the total number of pellets in a total volume of 1 mL organoid media or PBS. Resuspend thoroughly, washing the virus pellets from the UC tube bottoms.
    ***Note:*** If the virus is only to be used in the transduction of organoids, it can already be taken up in organoid medium. However, if other cells are also to be transduced with the virus, then uptake in PBS is recommended.
47. Store virus-containing aliquots at −80°C.

### Virus titer determination - Quantitative virus titer determination

**Timing: 15 min**

Determination of the lentiviral titer can be achieved in multiple ways and is dependent on your transgene. Transgene-independent measurement of lentiviral titers have been made possible by virus-protein detecting ELISAs or even faster by antigen tests like the 10-min Lenti-X GoStix Plus Tests (Takara). Here, the HIV p24 capsid protein is detected for quantifying the lentiviral titer.

***Note:*** Classic quantification of the MOI when expressing a fluorescent protein would be done by transducing cells and counting the cells that emit a fluorescent signal. This is time-consuming and unreliable for signaling-sensitive fluorophore expression transgenes or sensors.

48. Perform the test using the media supernatant of the viral particle production, revealing immediately whether the virus assembly has been successful. Carry out the application according to the manufacturer’s protocol.

**Pause point:** You can safely store the virus at −80°C for up to 1–2 years.

### Transduction of intestinal organoids with lentiviral particles

**Timing: 2 h organoid handling (+2 h pre-transduction and +6 h transduction time)**

Lentiviral transduction is an effective method to gain a stable and homogenous organoid line expressing a reporter gene for visualization of cellular processes involved in epithelial restitution. This requires the insertion of the transgene into the genome of the intestinal stem cells, as it can be achieved by the following step-by-step protocol, altered from De Van Lidth Jeude and colleagues from 2015.^25^

***Note:*** The intestinal organoids should be a low passage number (P3-P10) and two to three days post-passaging. Approximately 2–4 × 10^7^ IU of virus is required for the transduction of 6 wells of organoids.

49. Increase the susceptibility of the cell surface and virions.
  a. Incubate 6 wells of d2-d3 intestinal organoids with 500 μL/well organoid media supplemented with 8 μg/mL polybrene 2 h before transduction.
50. Precool the centrifuge, prewarm TrypLE Select (1×) at 37°C, and precoat 2 mL safe seal tube and 1.5 mL Eppendorf tube with 0.1% BSA in 1× PBS.
51. Remove the organoids from the extracellular matrix.
  a. Place the culture plate on ice to destabilize the extracellular matrix.
  b. Add 500 μL ice-cold 1× PBS to each well to detach the Cultrex-organoid droplets.
  c. Scratch the well bottom with the pipette tip and pipette the total volume of 1 mL up and down approx. 5 times to wash residual organoids from the well bottom and dissolve the matrix further.
  d. Collect the organoid-containing solution in a 15 mL canonical tube placed on ice.
  e. Wash the wells with 1 mL 1× PBS and transfer it into the 15 mL canonical tube.
52. Separate into single-cells.
  a. Centrifuge the organoid solution at 500 × *g* for 5 min at 4°C.
  b. Discard the supernatant; first by tilting the canonical tube and then removing the residual liquid using a 100 μL pipette.
    ***Note:*** Since you did not pull the organoid solution through a 24 G needle there will appear a milky layer of loosened extracellular matrix above the organoid pellet which you can carefully remove as well.
  c. Add 1 mL prewarmed TrypLE (1×) to the pellet and resuspend it.
  d. Place the tube at 37°C for 10 min with additional harsh mixing steps by pipetting (precoated P1000) every 2 min.
  e. Stop the fissuring of the peptide bonds by TrypLE with 4 mL organoid media and mix shortly. Transfer a small amount (approx. 500 μL) of the isolated organoid cell solution into a separate precoated canonical tube to be treated as a virus-negative control in the following steps.
  f. Centrifuge the single-cell solutions at 850 × *g* for 10 min at 4°C.
53. Transduce intestinal organoid cells with lentiviral particles.
  a. Take up the pellet in 1.5 mL transduction organoid media (see materials and equipment section), containing approx. 2–4 × 10^7^ IU of lentivirus, 8 μg/mL polybrene, 10 μM Y-27632, 100 ng/mL EGF and 2.5 μM CHIR99021.
  b. Transfer the organoid:virus solution to a 0.1% BSA-precoated 2 mL safe seal tube.

***Note:*** In the negative control, the volume of the virus is replaced with organoid medium.

54. Rotate the organoid:virus solution at 20 turnarounds/min at 4°C for 6 h, interrupt shortly by resuspending the cells by pipetting after 3 h.
55. Stop transduction and embed the organoid single-cells in the extracellular matrix.
  a. Transfer the organoid:virus mixture from the 2 mL into a 1.5 mL 0.1% BSA-precoated tube.
  b. Centrifuge at 850 × *g* for 10 min at 4°C.
  c. Discard the supernatant; first using a 1000 μL pipette and then a 100 μL pipette.
  d. Take up the pellet in Cultrex. Plan to plate half the number of wells with which this transduction was started, and 30 μL Cultrex/well.
  e. Seed 30 μL/well in three droplets and let them solidify for 20 min in an incubator at 37°C and 5% CO_2_.
  f. After 20 min add 500 μL prewarmed conditioned organoid media supplemented with additional 10 μM Y-27632 to each well and cultivate the organoid-forming cells at 37°C with 5% CO_2_.
56. Change media every two days. The increase in Y-27632 concentration from 10 to 20 μM is only maintained in the first two days after transduction.

***Note:*** The transduced intestinal organoids are S2 material right after transduction.

### Selection of successfully transduced intestinal organoids

The selection of successfully transduced intestinal organoids for regenerative studies can be done by the inserted resistance gene or, if the necessary sterility precautions are present, even by careful selection by hand (See Problem 6).

#### Selection by antibiotics

**Timing: 2–4 weeks**

***Note:*** Since the transduction of intestinal organoids takes place in their single-cell stadium, it takes about 1–2 weeks for them to generate fully grown colon organoids again. Start the antibiotic selection early on to obtain a high yield of positively transduced organoids.

***Note:*** You should start antibiotic substitution to the media right after passaging the transduced organoids and the selection pressure should be maintained until, at best, all organoids that do not emit a fluorescence signal under the microscope have become apoptotic and all treated organoids from the untransduced control have become apoptotic.

***Note:*** This can take up to a few weeks and rounds of passaging. It is best to establish a kill curve for each freshly prepared antibiotic stock.

57. Prepare a kill curve and select successfully transduced organoids by antibiotic treatment.
  a. Prepare at least 6 wells, if possible 12 wells for doublets, freshly passaged wt intestinal organoids – best generated from the same animal as the later lentivirally transduced organoids and at a similar passage number.
  b. Substitute the media with five different concentration steps of the antibiotics and leave one control untreated with antibiotics.
  c. Grow the organoids with media exchange every two days and evaluate which antibiotic concentration leads to death of the organoids compared to the control wells each day.
  d. Treat your transduced organoids with the antibiotic concentration at which the wt organoids have just about all died and the control has survived.

#### Selection by microscopic selection (only possible if sterility and safety precautions are present)

**Timing: 1–3 h**

58. Select successfully transduced organoids by hand under a microscope if appropriate sterility and safety precautions are present.
  ***Note:*** Advantageous when confronted with low transduction efficiency.
  a. Loosen the extracellular matrix droplets by placing the well plate on ice and adding 500 μL ice-cold 1× PBS into the wells.
  b. Cut a P1000 tip about 2–3 mm with sterile scissors.
  c. Scratch the well bottom with the cut P1000 tip slowly and pipette the total volume carefully up and down and dissolve the matrix further, trying to avoid the rupture of the organoids.
  d. Collect the organoid-containing solution and transfer it into a 35 mm glass-bottom dish (ibidi).
  e. Place the dish under the microscope and let the organoids settle to the glass bottom.
  f. Determine the positively transduced organoids by their fluorescence signal and collect the respective organoids in the bright-field mode with a P100 pipette tip.
  g. Transfer the organoids in 1× PBS on ice.
  h. Passage them as described in section passaging of murine colonic organoids.
  i. It is nevertheless recommended to carry out an antibiotic selection for at least two passages.
    **CRITICAL:** The antibiotic resistance genes are normally downstream of ubiquitous promoters, ensuring their expression is continuous during the growth of the organoids and extending the time frame for antibiotic selection. Selection by fluorescence emittance when the fluorescence gene is e.g. located downstream of a signaling-specific promoter requires attention to when the signaling of interest is active and the time that is needed for generating the fluorophore as well at its half-life.
    ***Note:*** There are automated alternatives available like the CellCelector Flex from Sartorius. However, these systems are associated with corresponding costs.

### Perform single-cell ablation in intestinal organoids using a femtosecond laser

**Timing: 30 min**–**2 h (depending on the number of organoids processed)**

#### Sample preparation

**Timing: setup: 5 min + 1–3 min/organoid**

59. Culture the samples in a 35 mm glass bottom dish (ibidi) for single-cell ablation.

***Note:*** Other dishes can also be used as long as the glass bottom is about the thickness of a cover slip.

***Note:*** When seeding, it is advised to seed the organoids close to the dish bottom by spreading the organoid containing Cultrex as flat as possible.

60. Turn on your femtosecond laser system and preheat and gas the incubation chamber to 37°C and 5% CO_2_ if available.
61. Set the wavelength and filters according to your fluorophore (e.g., 730 nm for green fluorophores, optical bandpass filter with transmission wavelength: 510–560 nm).

**Figure 3:**
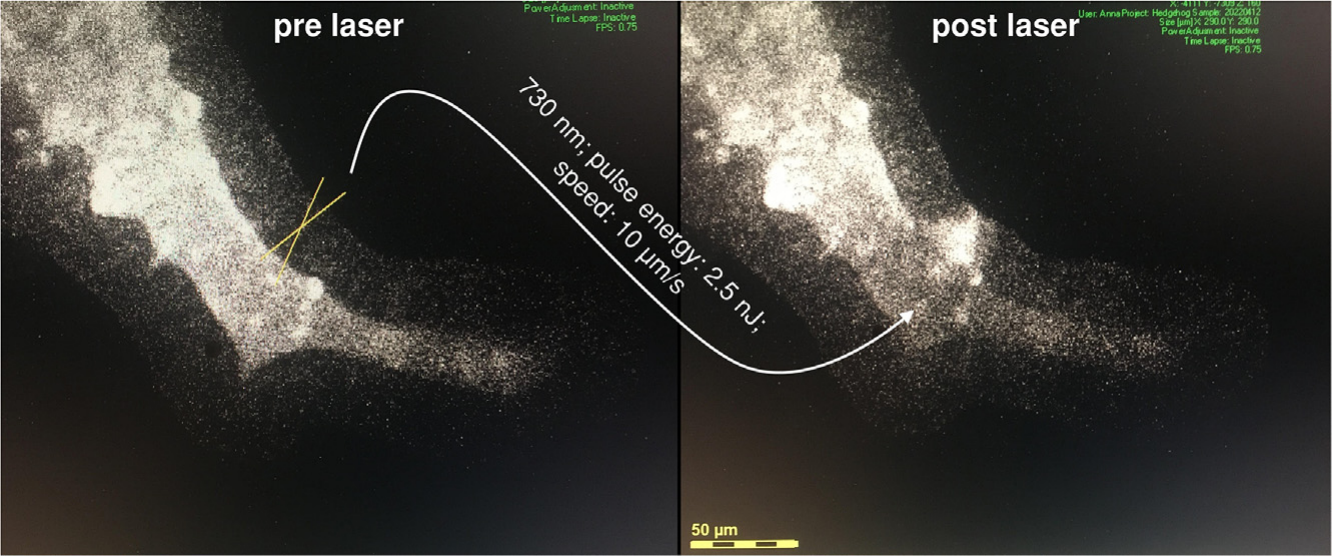
**Visualization of the cellular ablation process in a colonoid crypt by femtosecond laser using autofluorescence** The organoid crypt is imaged at 730 nm with the blue bandpass filter (460 ± 20 nm). The target site was marked with an X. The laser pulse power set to 2.5 nJ and the speed to 10 μm/s. The ablation site is clearly recognizable by an increase in the autofluorescence signal.

***Note:*** In case the chosen signal strength or distribution is not suited for the visualization of the epithelium, intestinal organoids depict NADH/FAD autofluorescence in the blue spectral range (Figure 3).

**Figure 4:**
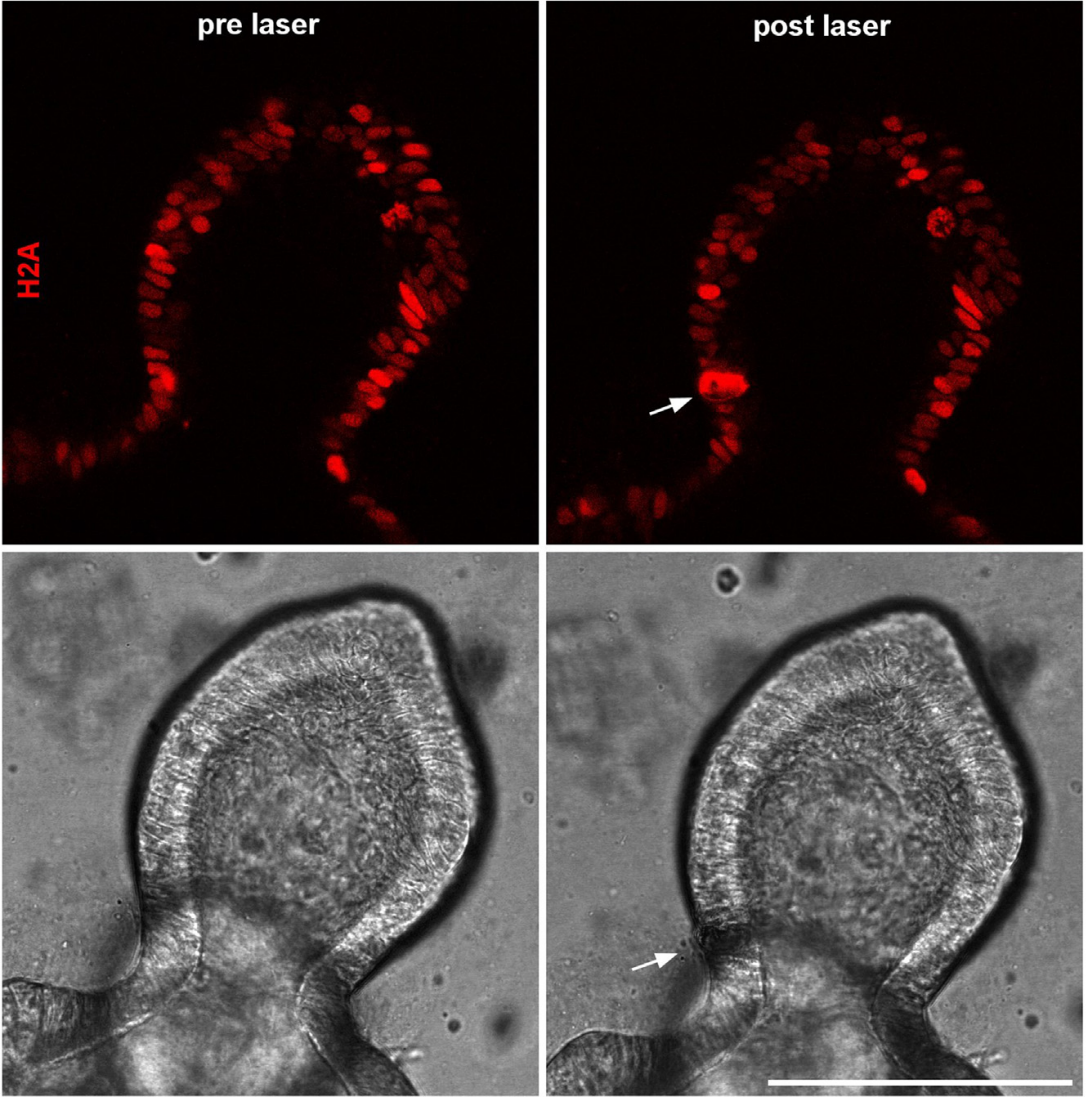
**Cellular ablation by femtosecond laser at the crypt neck of an organoid with the lentivirally introduced H2A-mCherry fusion protein** Confocal images of a H2A-mCherry-fusion protein expressing colon organoid before and after cellular ablation at the crypt neck. The figure shows one plane of the z-stack projected in Methods video S1. Arrow indicates the ablation site. Scale bar = 100 μm.

62. Adjust your laser power to 1–1.25 nJ for imaging and 1.25–3.2 nJ for single-cell ablation at the crypt base and up to 4.4 nJ for ablation in the differentiated epithelium (Figures 3 and 4).

**CRITICAL:** Due to the individuality of the organoids’ position and differentiation state it is always advised to start at the lower limit of your power range and approach the needed power for ablation carefully. If the laser power for cell ablation needs to be adjusted, it is advised to increase the laser power in 0.125 nJ steps.

**CRITICAL:** Be aware that the PMT should never be exposed to strong light (room light, bright-field lamp).

#### Cell ablation by femtosecond laser

**Timing: setup: 5 min + 1–3 min/organoid**

63. Place your sample dish so the organoid of interest is in focus and positioned as centrally as possible.
64. Aim at the cell of interest by drawing an X (several μm) as a laser line that does not pass the neighboring cells, but runs across the apical and basolateral cell borders.
65. Ablate the cell at a laser scanning speed of 7–10 μm/s (See Problem 8).
66. Successful ablation is visible by an increase in autofluorescence at the ablation site (Figures 3 and 4, Methods video S1; See also Problem 7).

67. Either image the process of interest directly using two-photon microscopy or switch to the confocal microscope as described in the following section (Figure 4, Methods video S1).

Methods video S1. Visualization of femtosecond laser-induced cellular ablation in the crypt of a H2A-mCherry colon organoid, related to section 'Cell ablation by femtosecond laser' The video shows the same crypt of a colon organoid successfully transduced with a lentivirus integrating the H2A-mCherry fusion protein before and after femtosecond laser-based cellular ablation. Confocal images. The z-stack (50 μm depth, z-step size 4 μm) was converted into a 3D projection using the brightest point projection method and interpolation, with 15 fps. The circle marks the ablation site. Scale bar = 100 μm.

### Imaging of dynamic cellular processes in intestinal organoids

**Timing: varying**

Signaling processes are highly dynamic and their visualization can therefore be challenging, especially when aiming for spatiotemporal resolution of the modulators of intestinal regeneration. Since the fluorophore is expressed by the cells themselves, the imaging does not require any staining steps (except in the case of small peptide tags) and can be performed in live cells during regeneration and over longer periods of time and generally with every commercial fluorescence microscope with high axial resolution for 3D imaging.

***Note:*** This protocol was performed using the Leica TCS SP5 confocal microscope together with the Leica Application Suite AF software. The 25× 0.95 NA water-immersion objective is preferred for image acquisition due to its high light yield. A PCO Edge 4.2 camera is installed for further wide-field signal detection. The necessary excitation wavelength depends on the utilized fluorophore. The microscope is equipped with an incubation chamber allowing controlled surroundings of 37°C and 5% CO_2_.

***Note:*** If developmental events are to be followed closely over the course of several days, grid dishes are helpful to ensure that an organoid can be identified again.

***Note:*** Remember necessary controls, for example untreated organoids in an extra 35 mm glass bottom dish if the organoids are treated with additional chemicals that may have an influence on the process under investigation.

68. Prepare your imaging equipment.

***Note:*** When imaging the cellular process visualized by the transgene fluorophore over a longer period of time, the imaging equipment should provide surroundings of 37°C and 5% CO_2_.

69. Set your imaging parameters (e.g., laser power, gain, and pinhole width) in accordance to sample (transduced organoid) and negative control (wt/untreated organoid).

***Note:*** Organoids and the extracellular matrix can emit high background signals, caused by collagen and elastin fibers and organelles such as mitochondria and lysozymes, so setting the right imaging parameters is crucial for correct identification of your target signal.^26^^,^^27^

**CRITICAL:** If an ablation process is also to be carried out using TPEM, it must be considered that the ablation site may depict high levels of autofluorescence in the green spectra, as the metabolic activity decreases and cellular contents leak from the cell, as e.g. pyridine nucleotides like NADH.^28^^,^^29^

70. Acquire your images.
  a. Take Z-stacks to obtain the full volume of the organoid while keeping localization information intact (Methods video S1).

***Note:*** For an overview, a 10 μm step size is sufficient. For cellular resolution, 2–4 μm step size is required. The acquisition of z-stacks takes time and might cause photobleaching. If the fluorescence signal of interest changes quickly, for example in case of calcium-dependent signals, a plane of interest should be determined and only imaged +/− 4 μm above and below from the plane.

***Note:*** Detection of multichannel fluorescence can be realized with sequential excitation to avoid overlapping of fluorescence between channels.

**Figure 5:**
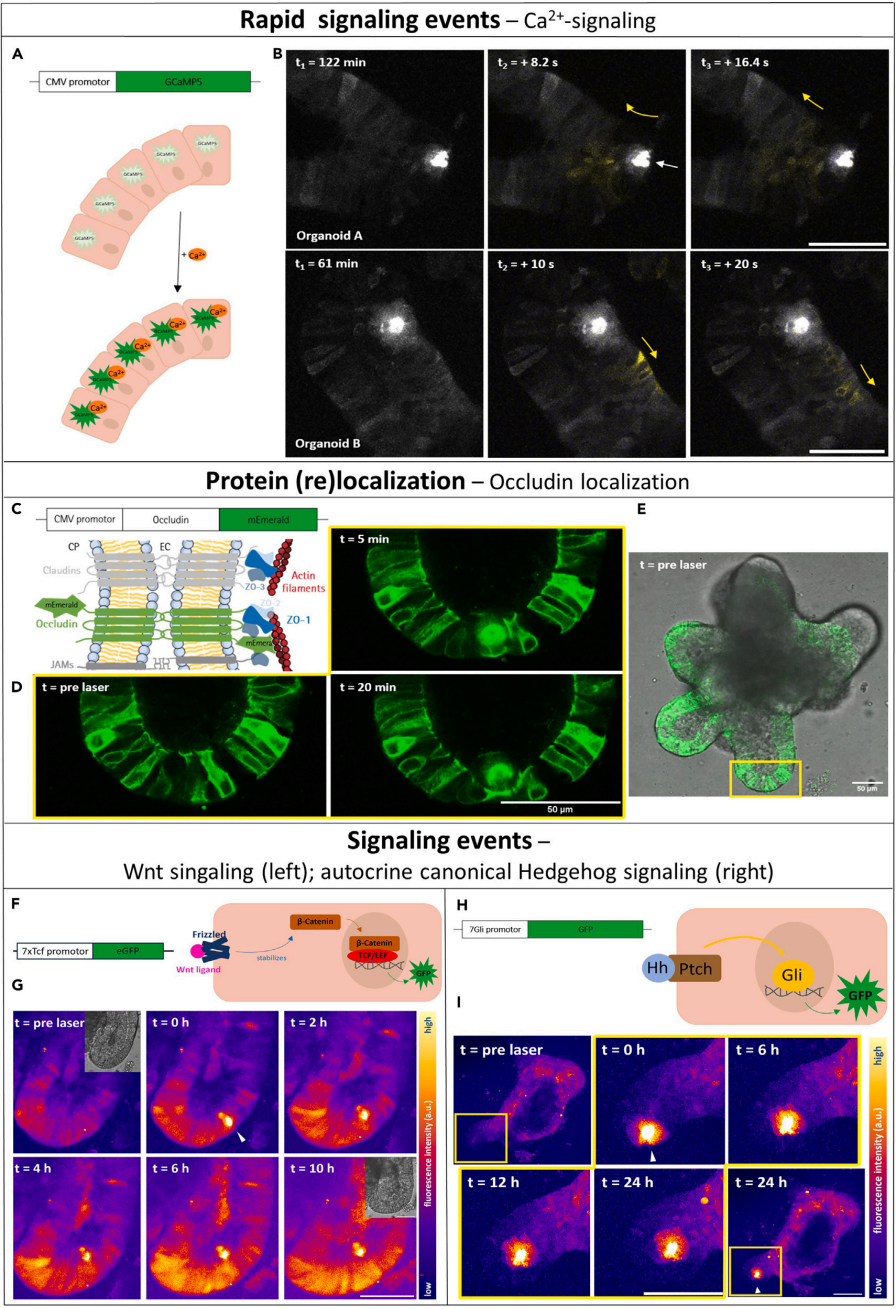
**Four examples of possible applications of fluorescent biosensors for the investigation of the epithelial regeneration process in organoids after femtosecond laser-induced single-cell ablation** (top) Calcium waves in lentivirally transduced CMV-GCaMP5 colon organoids after laser ablation. (A) Schematic representation of free intracellular Ca^2+^ detection by the GCaMP5 indicator. (B) Intracellular calcium waves propagating from the wound sites during the regeneration process in GCaMP5-expressing colon organoids. Crypts are shown as z-projections (confocal microscopy, (Organoid A) 9 μm depth, z-step size 3 μm, (Organoid B) 12 μm depth, z-step size 3 μm; max intensity of green fluorescence signal). Scale bar = 50 μm. For details refer to Donath et al., 2023.^2^ (middle) Occludin accumulation at the wound-closing cell membranes visualized by fusion protein Occludin-mEmerald as an example for the tracking of protein localizations during the epithelial restitution after single-cell ablation by femtosecond laser. (C) Illustration of a tight junction with fluorescent fusion protein Occludin-mEmerald. (D) Zoomed sections of the laser-ablation site show formation of “Occludin-ring” during epithelial restitution and exfoliation (0–20 min post laser-damage). Confocal images. (E) Merged bright-field and fluorescence image of CMV-Occludin-mEmerald transduced colonoid, overview. Scale bar = 50 μm. (bottom, left box) Increased canonical Wnt signaling in wound neighboring cells after femtosecond laser-based cell ablation. (F) Schematic representation of how the Wnt signaling-sensitive 7xTcf promoter construct works. (G) Time course (0 h–10 h post laser ablation) of Wnt signaling activity during the regeneration process of ablation damage at the crypt base of a 7xTcf-promoter-EGFP transduced colon organoid. Increase of the Wnt-dependent fluorescence signal at the crypt base with time. Images depict z-projections (confocal microscopy, 52 μm depth, z-step size 2 μm, max intensity of green fluorescence signal, lookup table fire). Scale bar = 50 μm. For further information and control crypt view Donath et al.^1^ (bottom, right box) Autocrine canonical hedgehog signaling is not involved in the repair of laser-induced damage to the proliferative zone at the crypt base in murine colon organoids. (H) Schematic representation of the Gli-dependent EGFP expression visualizing canonical hedgehog signaling. (I) Overview of a lentivirally 7Gli-promoter-EGFP transduced colon organoid pre and 24 h post wound infliction by femtosecond laser at the crypt base. (I, yellow bordered) Zoomed sections of the ablation site show the regeneration of the crypt epithelium within 24 h without shift or changes in GFP signal of the canonical hedgehog signaling. Colon organoid overview and the crypt close ups are shown as z-projections (confocal microscopy, 34 μm depth, z-step size 2 μm, max intensity of green fluorescence signal, lookup table fire). Arrow indicates the ablation site. Representative images of *n* = 8; 4 technical replicates. Scale bar = 100 μm.
  b. Select 15–60 min imaging intervals for medium-speed processes (e.g., changes in protein localization, Figures 5C–5E); medium imaging volume (about 10–200 μm); medium line of frame averaging (2–6). Total recording period up to a few hours. As organoids show strong cellular movements, adaptations of the focus planes will be needed.
  c. Pick imaging intervals of 30–120 min to 24 h for slow processes (e.g., Wnt or Hedgehog-signaling during epithelial regeneration or development, Figures 5F–5I).
    ***Note:*** The first when tracking cellular distribution or changes in strength of the signal of interest during a process like epithelial regeneration and the latter when being interested in signaling or expressional changes during long-term development; larger imaging volumes; high frame or line averaging possible. The focus can be on high-resolution imaging.
    **CRITICAL:** Constant imaging of long developmental processes is not recommended by confocal microscopy due to fluorophore bleaching, but other techniques such as light sheet microscopy or wide-field microscopy.
72. Integrate your phototoxicity control, which are wt/untreated organoids that are exposed to the same imaging process and assessed for their well-being and/or fluorescence signal.

***Note:*** Since *in vivo* imaging not only influences the lifetime of the fluorophore, but also the well-being of the organoid sample, it is important to perform a phototoxicity control.

71. Select the appropriate time interval between images.
  ***Note:*** The chosen time interval between the image acquisition can be critical and depends on your process of interest, your fluorophore and image equipment, and vice versa does the imaging interval determine your fluorophore live time. Settings should always be selected to match the question and cause as little phototoxicity damage as possible to organoids and fluorophores.
  a. Choose short intervals between image acquisition of 1–60 s when you image fast processes (e.g., Calcium signaling, Figures 5A and 5B); small total imaging volume (about 5–20 μm, small step size); low line or frame averaging (0–2); low laser power. Total recording period of maximal few hours recommended.
    **CRITICAL:** Due to its spot-by-spot scanning, the confocal microscope quickly reaches its limits when it comes to rapidly capturing many images of larger volumes. In this case, techniques such as light sheet microscopy or wide-field microscopy are more suitable.

### Imaging analysis

**Timing: varying**

The resulting image data sets can become very large, especially with confocal images of different planes over long periods of time. In addition, it is always a challenge to be as objective as possible when analyzing image data. For this reason, artificial intelligence tools, often developed by other scientists who have faced similar challenges, are helpful for analyzing image data.

Example: Analysis of cell morphology – cell area, perimeter, aspect ratio - using Occludin-mEmerald expressing colon organoids.^2^

73. Install Cellpose 2.0 for 2D cell segmentation.

***Note:*** Cellpose is a pre-trained deep-learning network for cell and nucleus segmentation. The algorithm relies on the manual labeling followed by a learning phase reproducing the annotations and allows the revision of the automatically generated results.^16^

74. Split the Z-stacks into planes or use the 3D stack for Cellpose. Depending on your imaging and cell parameters, try different pre-trained models and optimize them by further cell labeling until you reach sufficient cell identification.

***Note:*** For example, we trained a custom model based on the cyto cellpose model successfully using 100 epochs in each training step. Segmentation cell diameter was 45 pixel.

75. Run Cellpose on your imaging data to receive “Label Images”, which can be analyzed in further processing steps.
76. Use an ImageJ plugin such as LabelsToROIs to convert Cell Pose Label Images to images containing ROIs.^30^ These can then be further analyzed using ImageJs integrated analysis functions. Consider writing your own macro code to perform these tasks automatically.
77. Map the collected data to a summarizing parameter such as the distance to the ablation site in μm and by analyzed using statistic software like GraphPad or OriginPro 2019 (OriginLab Corporation, Northampton, MA, USA).

***Note:*** There are alternatives to ImageJ like for example Napari or MoBIE which can be used for image visualization and analysis.

## Expected outcomes

This protocol provides you with the step-by-step instructions from the cultivation of colonic organoids to imaging of dynamic processes during wound healing of the murine intestinal epithelium after femtosecond laser-induced wounding. The crypt harvest from one mouse should result in 500–900 viable, crypt-forming organoids, depending on the colon size and crypt release. These numbers can even be multiplied by further passaging or long-term stored by cryopreservation. Accordingly, one crypt isolation run is sufficient for many transduction trials. The transduction efficiency is highly dependent on the virus and introduced sequence but we have found that an efficient transduction results in 2%–30% transduced organoids, and by propagating the organoids through cultivation, the planned experiments can be carried out in full. Accordingly, in theory, one animal is sufficient to answer multiple questions regarding the process of wound healing. However, for the transferability of the results, consideration should be given to conducting the experiments on multiple genetic backgrounds. The laser ablation method is reliable and can be used for a wide range of applications; ablation at defined times and precise locations, even subcellular structures. These protocols enable a wide range of questions regarding regeneration processes in the intestinal epithelium to be answered with minimal animal consumption by visualizing the processes involved.

## Limitations

In general, the use of organoids is associated with limitations compared to standard monoclonal cell cultures: The genetic background of mouse organoids is more variable and is often isolated from several animals. The complexity regarding cellular interactions is increased compared to monoclonal cell culture by the generation of different cell types of the intestinal epithelium, however physiological interaction partners such as stromal cells or immune cells as they are present *in vivo* are neglected.

Additionally, the utilization of lentiviruses for genetic modification always involves the downside that the number of transgene insertion events and their localization in the genome is uncontrolled. This is also the reason why quantification of the fluorescence data can only be carried out, when there is a reference fluorophore that is downstream of the same promoter and independent of your process of interest and after thorough calibration. It is also important to differentiate which form of wounding and repair response you want to imitate. In our setup, laser ablation is used to recreate ‘open wounds’ very abruptly, probably recreating a necrotic state. Literature shows that the femtosecond laser setup can also be used for apoptosis induction if preferred.^31^^,^^32^ However, this is of course not a suitable model for imitating inflammation-induced apoptosis, for example.

## Troubleshooting

### Problem 1

The colon organoids cannot be mechanically fragmented sufficiently by the 24G needle into single crypts.

### Potential solution

You can try to increase the number or pressure of pulling the organoids through the 24G needle. However, you don’t want the colon organoids to separate into single cells or cluster of cells. Another solution would be to remove the organoids from the Cultrex as described and take them up in 1 mL TrypLE Select (1×) after centrifugation, place them at 4°C for 2 min before pipetting the solution up and down 2–3 times and inactivating the TrypLE Select (1×) with organoid media. Centrifuge again with 750 × *g* at 4°C for 5 min and continue as stated in the protocol passaging of murine colonic organoids step 2.c.

### Problem 2

Colon organoids are not evenly distributed in the Cultrex droplet, but rather accumulated in the bottom layer. Or the organoids tend to adhere and grow on the bottom of the well or dish.

### Potential solution

To prevent the organoids from partially growing on the well or dish bottom, it can help to turn the plate or dish upside down 1 min after plating the droplets. Keep them upside down until the Cultrex is solidified and the medium added.

### Problem 3

Reaction conditions of the restriction enzymes for lentiviral backbone digestion are not compatible.

### Potential solution

When the restriction enzymes needed for the transgene insertion sites are not compatible regarding their reaction conditions, it is possible to perform the reactions for each restriction enzyme in succession and use the Monarch PCR&DNA Cleanup Kit (New England Biolabs) in between.

### Problem 4

Unsuccessful amplification of inserts due to premature termination of synthesis or occurrence of mutations.

### Potential solution

Use a polymerase with proofreading quality and optimized for amplification of long transcripts, e.g., the GoTaq Long PCR Master Mix from Promega. And recheck the compatibility of your primer pairs. You may need to re-plan your cloning with new primers.

### Problem 5

Unsuccessful HiFi Assembly of the cut lentiviral backbone and the transgene fragment(s).

### Potential solution

First, we recommend that you extend the HiFi reaction by carrying out the assembly reaction 12–16 h at 50°C. In case of reassembly of the lentiviral backbone with the originally excised fragment, it may help to excise the band of the excised backbone from the agarose gel and purify it for the HiFi reaction.

### Problem 6

Low transduction efficiency.

### Potential solution

Low transduction efficiency can have many causes. However, before discarding the virus and designing new transfer plasmids, we recommend testing the virus in easy-to-transduce cells such as HEK293 or HeLa (making sure that the promoter is active in these cells) and/or increase the virus titer used for transducing the organoid cells.

### Problem 7

The ablation process does not take place even though sufficient laser power has been used.

### Potential solution

If it should happen that the organoid is difficult to focus or if successful ablation is not possible even after increasing the laser power, this may be caused by an elevated position of the organoid in the extracellular matrix. By carefully removing the organoid from the matrix and re-embedding it in (diluted) Cultrex. Let the Cultrex solidify at 20°C–25°C for 5 min before placing it at 37°C. It may be possible to place it nearer the glass bottom of the 35 mm glass bottom dish (ibidi).

### Problem 8

During the ablation process, not just one but several cells are ablated. Or cavitation bubbles are formed during ablation.

### Potential solution

These are clear signs that too much laser power is being used or your target is selected too unspecific. Decrease the power to 1 nJ and increase it in 0.125 nJ increments. If necessary, you can mark the cell for ablation without going over the cellular edges.

## Resource availability

### Lead contact

Further information and requests for resources and reagents should be directed to and will be fulfilled by the lead contact, Stefan Kalies, kalies@iqo.uni-hannover.de.

### Technical contact

Questions about the technical specifics of performing the protocol should be directed to and will be answered by the technical contact, Stefan Kalies, kalies@iqo.uni-hannover.de.

### Materials availability

This study did not generate new unique reagents.

### Data and code availability

This study did not generate/analyze new data or code sets. Please refer to the referenced publications.

## References

[1] Donath S., Angerstein L., Gentemann L., Müller D., Seidler A.E., Jesinghaus C., Bleich A., Heisterkamp A., Buettner M., Kalies S. (2022). Investigation of Colonic Regeneration via Precise Damage Application Using Femtosecond Laser-Based Nanosurgery. Cells.

[2] Donath S., Seidler A.E., Mundin K., Wenzel J., Scholz J., Gentemann L., Kalies J., Faix J., Ngezahayo A., Bleich A. (2023). Epithelial restitution in 3D - Revealing biomechanical and physiochemical dynamics in intestinal organoids via fs laser nanosurgery. iScience.

[3] Gentemann L., Donath S., Seidler A.E., Patyk L., Buettner M., Heisterkamp A., Kalies S. (2023). Mimicking acute airway tissue damage using femtosecond laser nanosurgery in airway organoids. Front. Cell Dev. Biol..

[4] Teschendorf C., Warrington K.H., Siemann D.W., Muzyczka N. (2002). Comparison of the EF-1 alpha and the CMV promoter for engineering stable tumor cell lines using recombinant adeno-associated virus. Anticancer Res..

[5] Das A.T., Tenenbaum L., Berkhout B. (2016). Tet-On Systems For Doxycycline-inducible Gene Expression. Curr. Gene Ther..

[6] Liu Z., Chen O., Wall J.B.J., Zheng M., Zhou Y., Wang L., Vaseghi H.R., Qian L., Liu J. (2017). Systematic comparison of 2A peptides for cloning multi-genes in a polycistronic vector. Sci. Rep..

[7] Shaner N.C., Lambert G.G., Chammas A., Ni Y., Cranfill P.J., Baird M.A., Sell B.R., Allen J.R., Day R.N., Israelsson M. (2013). A bright monomeric green fluorescent protein derived from Branchiostoma lanceolatum. Nat. Methods.

[8] Ivorra-Molla E., Akhuli D., McAndrew M.B.L., Scott W., Kumar L., Palani S., Mishima M., Crow A., Balasubramanian M.K. (2023). A monomeric StayGold fluorescent protein. Nat. Biotechnol..

[9] Gadella T.W.J., van Weeren L., Stouthamer J., Hink M.A., Wolters A.H.G., Giepmans B.N.G., Aumonier S., Dupuy J., Royant A. (2023). mScarlet3: a brilliant and fast-maturing red fluorescent protein. Nat. Methods.

[10] Machleidt T., Robers M., Hanson G.T. (2007). Protein labeling with FlAsH and ReAsH. Methods Mol. Biol..

[11] Provost C.R., Sun L. (2010). Fluorescent Labeling of COS-7 Expressing SNAP-tag Fusion Proteins for Live Cell Imaging. J. Vis. Exp..

[12] Tanenbaum M.E., Gilbert L.A., Qi L.S., Weissman J.S., Vale R.D. (2014). A Protein-Tagging System for Signal Amplification in Gene Expression and Fluorescence Imaging. Cell.

[13] Beck E., Ludwig G., Auerswald E.A., Reiss B., Schaller H. (1982). Nucleotide sequence and exact localization of the neomycin phosphotransferase gene from transposon Tn5. Gene.

[14] Fuerer C., Nusse R. (2010). Lentiviral Vectors to Probe and Manipulate the Wnt Signaling Pathway. PLoS One.

[15] Dull T., Zufferey R., Kelly M., Mandel R.J., Nguyen M., Trono D., Naldini L. (1998). A Third-Generation Lentivirus Vector with a Conditional Packaging System. J. Virol..

[16] Stringer C., Wang T., Michaelos M., Pachitariu M. (2021). Cellpose: a generalist algorithm for cellular segmentation. Nat. Methods.

[17] Schindelin J., Arganda-Carreras I., Frise E., Kaynig V., Longair M., Pietzsch T., Preibisch S., Rueden C., Saalfeld S., Schmid B. (2012). Fiji: an open-source platform for biological-image analysis. Nat. Methods.

[18] Sato T., Vries R.G., Snippert H.J., van de Wetering M., Barker N., Stange D.E., van Es J.H., Abo A., Kujala P., Peters P.J., Clevers H. (2009). Single Lgr5 stem cells build crypt-villus structures in vitro without a mesenchymal niche. Nature.

[19] Clevers H. (2013). The Intestinal Crypt, A Prototype Stem Cell Compartment. Cell.

[20] Mahe M.M., Aihara E., Schumacher M.A., Zavros Y., Montrose M.H., Helmrath M.A., Sato T., Shroyer N.F. (2013). Establishment of Gastrointestinal Epithelial Organoids. Curr. Protoc. Mouse Biol..

[21] Gill K.P., Denham M. (2020). Optimized Transgene Delivery Using Third-Generation Lentiviruses. Curr. Protoc. Mol. Biol..

[22] De Duve C., De Barsy T., Poole B., Trouet A., Tulkens P., Van Hoof F. (1974). Commentary. Lysosomotropic agents. Biochem. Pharmacol..

[23] Pinto-González Howell D., Krieser R.J., Eastman A., Barry M.A. (2003). Deoxyribonuclease II is a lysosomal barrier to transfection. Mol. Ther..

[24] Ohkuma S., Poole B. (1978). Fluorescence probe measurement of the intralysosomal pH in living cells and the perturbation of pH by various agents. Proc. Natl. Acad. Sci. USA.

[25] De Van Lidth Jeude J.F., Vermeulen J.L.M., Montenegro-Miranda P.S., Van Den Brink G.R., Heijmans J. (2015). A protocol for lentiviral transduction and downstream analysis of intestinal organoids. JoVE.

[26] Jenvey C.J., Stabel J.R. (2017). Autofluorescence and Nonspecific Immunofluorescent Labeling in Frozen Bovine Intestinal Tissue Sections: Solutions for Multicolor Immunofluorescence Experiments. J. Histochem. Cytochem..

[27] Dacosta R.S., Andersson H., Cirocco M., Marcon N.E., Wilson B.C. (2005). Autofluorescence characterisation of isolated whole crypts and primary cultured human epithelial cells from normal, hyperplastic, and adenomatous colonic mucosa. J. Clin. Pathol..

[28] Chance B., Legallais V., Schoener B. (1962). Metabolically Linked Changes in Fluorescence Emission Spectra of Cortex of Rat Brain, Kidney and Adrenal Gland. Nature.

[29] Anderson C.D., Lin W.C., Beckham J., Mahadevan-Jansen A., Buttemere C.R., Pierce J., Nicoud I.B., Wright Pinson C., Chari R.S. (2004). Fluorescence spectroscopy accurately detects irreversible cell damage during hepatic radiofrequency ablation. Surgery.

[30] Waisman A., Norris A.M., Elías Costa M., Kopinke D. (2021). Automatic and unbiased segmentation and quantification of myofibers in skeletal muscle. Sci. Rep..

[31] Okano K., Wang C.-H., Hong Z.-Y., Hosokawa Y., Liau I. (2020). Selective induction of targeted cell death and elimination by near-infrared femtosecond laser ablation. Biochem. Biophys. Rep..

[32] Hill R.A., Damisah E.C., Chen F., Kwan A.C., Grutzendler J. (2017). Targeted two-photon chemical apoptotic ablation of defined cell types in vivo. Nat. Commun..

