# Supplementary material for: Protocol for the application of single-cell damage in murine intestinal organoid models
Source: STAR Protoc. 2024 Jul 30;5(3):103153. doi: 10.1016/j.xpro.2024.103153 (PMC11342180; doi:10.1016/j.xpro.2024.103153)
Supplement: Document S1. Figure S1 [file mmc1.pdf]

Supplemental information

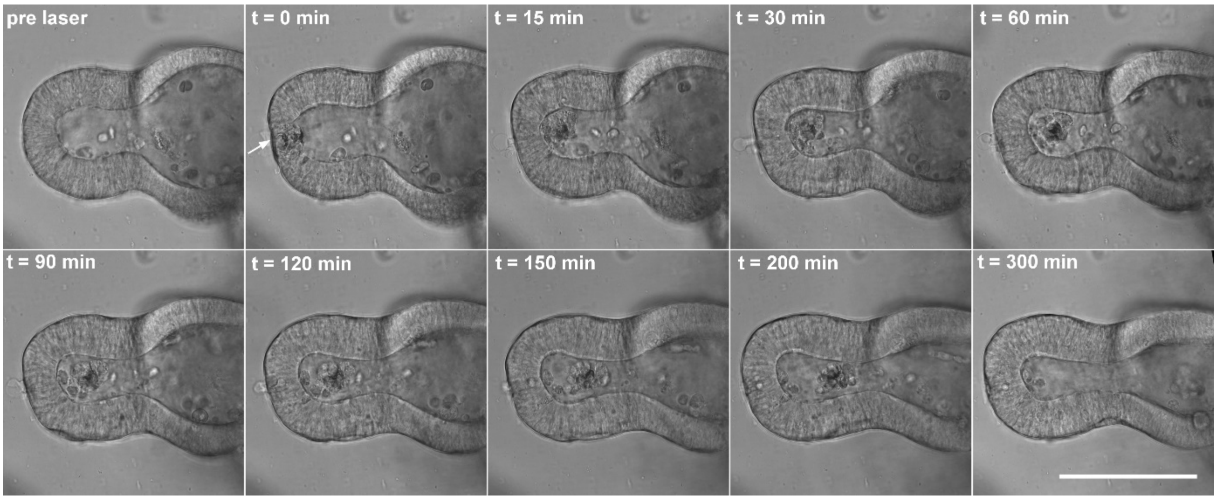

Figure S1: Epithelial repair after femtosecond laser-induced cell ablation at the crypt base of murine ileal organoids, related to section 'Before you begin'. Organoids were isolated from the distal ileum of C57BL/6J mice. A single cell was targeted with the femtosecond laser set to 730 nm and a pulse energy of 1.9 nJ. The epithelium is visually intact 15 min after ablation and the cell debris of the ablation site is transported towards the lumen in the following 5 h. Times of image acquisition is indicated in the upper left corners of the images. Bright-field images. The arrow indicates the ablation site. Scale bar = 100 µm.
